# Structure-based targeting of the lipid A-modifying enzyme PmrC to contrast colistin resistance in *Acinetobacter baumannii*

**DOI:** 10.3389/fmicb.2024.1501051

**Published:** 2024-11-28

**Authors:** Maria Romano, Federico Falchi, Eliana De Gregorio, Maria Stabile, Antonella Migliaccio, Alessia Ruggiero, Valeria Napolitano, Ida Autiero, Flavia Squeglia, Rita Berisio

**Affiliations:** ^1^Department of Biomedical Sciences, Institute of Biostructures and Bioimaging, National Research Council (CNR), Napoli, Italy; ^2^Department of Pharmacy and Biotechnology, Alma Mater Studiorum—University of Bologna, Bologna, Italy; ^3^Computational and Chemical Biology, Italian Institute of Technology, Genova, Italy; ^4^Department of Molecular Medicine and Medical Biotechnology, University of Naples Federico II, Naples, Italy; ^5^Department of Public Health, University of Naples Federico II, Naples, Italy

**Keywords:** protein structure, colistin resistance, virtual screening, pEtN transferase, cell wall

## Abstract

**Introduction:**

Antimicrobial-resistant pathogens are an ongoing threat to human and animal health. According to the World Health Organization (WHO), colistin is considered the last resort antibiotic against human infections due to multidrug-resistant Gram-negative organisms—including *Acinetobacter baumanni*, a priority-1 pathogen. Despite colistin being considered a last resort antibiotic, transferable bacterial resistance to this drug has been reported in humans and animals. This makes addressing colistin resistance a critical priority in public health efforts. The large PetN transferase membrane protein PmrC is responsible for colistin resistance due to its catalysed modification of lipid A of the external membrane. Despite its importance, this potential drug target was never characterised at a molecular level.

**Methods:**

The recombinant production of large membrane proteins in their native forms is a bottleneck in modern molecular biology. In this study, we recombinantly produced PmrC and biophysically characterised it in solution. We employed *in silico* approaches, including virtual screening and molecular modelling, to identify PmrC ligands. The binding of these ligands to PmrC was measured using Microscale Thermophoresis (MST). The best ligand was tested for its ability to hamper colistin resistance in *Acinetobacter baumannii* clinical isolates. Finally, we checked that the identified compound was not cytotoxic at the used concentrations by haemolysis assays.

**Results:**

We successfully produced PmrC PetN transferase membrane protein in high yields and showed that PmrC is a stable α-β protein, with melting temperature T_m_ = 60°C. Based on the PmrC structural model, we identified a promising druggable cavity. Therefore, we used a structure-based virtual screening to identify potential inhibitors. A small molecule, here denominated as s-Phen, was proved to bind PmrC with μM affinity. Microbiological assays confirmed that the s-Phen can drastically reduce colistin minimum inhibitory concentration (MIC) in two *A. baumannii*-resistant isolates and that it is not cytotoxic. Importantly, PmrC binding pocket to s-Phen is highly conserved in all homologues of PmrC, regardless of the location of genes encoding for them and of their operons.

**Discussion:**

Our study provides a molecular characterisation of PmrC and demonstrates the importance of PmrC as a drug target and the strong potential of PmrC binding molecules to act as colistin adjuvants, operating as synergistic tools to combat multiresistant nosocomial pathogens.

## 1 Introduction

The ESKAPE group of pathogens (*Enterococcus faecium*, *Staphylococcus aureus*, *Klebsiella pneumoniae*, *Acinetobacter baumannii*, *Pseudomonas aeruginosa*, and *Enterobacter* spp.) are responsible for complicated nosocomial infections worldwide and are often resistant to commonly used antibiotics in clinical settings (Kramarska et al., 2024; Sadones et al., 2024). Among ESKAPE, *A. baumannii* is an opportunistic Gram-negative coccobacillus that was declared by the World Health Organization (WHO) as among the most critical, owing to its formidable adaptability and resistance to antimicrobial therapies. Indeed, *A. baumannii* has progressively become resistant to many antibiotics (multidrug-resistant *A. baumannii* [MDR] *A. baumannii*), including carbapenems and third-generation cephalosporins, thus becoming a burden on healthcare facilities owing to limited treatment options (Karakonstantis et al., 2020; Oyejobi et al., 2022; Zampaloni et al., 2024).

Currently, colistin (also known as polymyxin E) is prescribed as a last resort for the treatment of MDR Gram-negative infections, including *A. baumannii* (Seleim et al., 2022; Zafer et al., 2023). The bactericidal activity of colistin, a positively charged peptide, relies on its ability to bind through electrostatic interactions to the negatively charged phosphate groups of lipid A, an important component of lipopolysaccharide (LPS). Colistin binding competitively displaces divalent cations from the phosphate groups of membrane lipids, leading to the disruption of the outer cell membrane (Cafiso et al., 2018). Although colistin is considered by the European Medicines Agency (EMA) as critically important in human medicine, resistance in clinical *A. baumannii* isolates is troublingly increasing due to its use in multiresistant forms (Hashemi et al., 2019).

Two primary mechanisms that provide colistin resistance have been described in *A. baumannii*: the complete loss of LPS or its modifications, which lead to the reduction of its negative charge (Hamel et al., 2021; Novović and Jovčić, 2023; Olaitan et al., 2014). In addition, plasmid-mediated colistin resistance encoded by mobilised colistin resistance (mcr) genes is a driver of rapid dissemination by horizontal gene transfer among pathogenic Gram-negative bacteria, including *A. baumannii* (Novović and Jovčić, 2023). The modification of LPS occurs by adding phosphoethanolamine (PetN) cationic groups to the lipid A. In several Gram-negative pathogens (*Salmonella enterica*, *K. pneumoniae*, *Escherichia coli*, and *P. aeruginosa*), another modification of LPS, the addition of the 4-amino-4-deoxy-l-arabinose (L-Ara4N), has been described as a more effective colistin resistance mechanism, compared to the addition of PetN (Olaitan et al., 2014). However, Ara4N biosynthesis and attachment genes are not present in *A. baumannii*, which suggests that Ara4N modification of lipid A is not suitable to explain colistin resistance in this bacterium (Novović and Jovčić, 2023; Olaitan et al., 2014). The complete loss of LPS results from inactivation of the first three genes of the lipid A biosynthetic pathway (lpxA, lpxC, and lpxD genes). This mechanism ensures a high level of colistin resistance (Moffatt et al., 2010). However, the frequency of mutations in the lpxACD is lower compared to changes in the operons encoding for PetN transferases in colistin-resistant *A. baumannii* clinical isolates (Kamoshida et al., 2020; Karakonstantis et al., 2020). The resulting lower prevalence of LPS-deficient colistin-resistant mutants in clinical settings is likely due to the significant negative impact of LPS loss on fitness, virulence, and susceptibility of these isolates to various antibiotics. Indeed, a strongly increased susceptibility to various clinically used antibiotics used in the therapy of *A. baumannii* infections (e.g., ceftazidime, imipenem, meropenem, tigecycline, ciprofloxacin, amikacin, and rifampin), and various disinfectants (e.g., quaternary ammonium disinfectants, sodium dodecyl sulfate, benzalkonium, and chlorhexidine) as well as to neutrophil-secreted lysozyme has been observed (Kamoshida et al., 2020; Karakonstantis et al., 2020; Moffatt et al., 2010; Oyejobi et al., 2022; Zampaloni et al., 2024).

**Figure 1:**
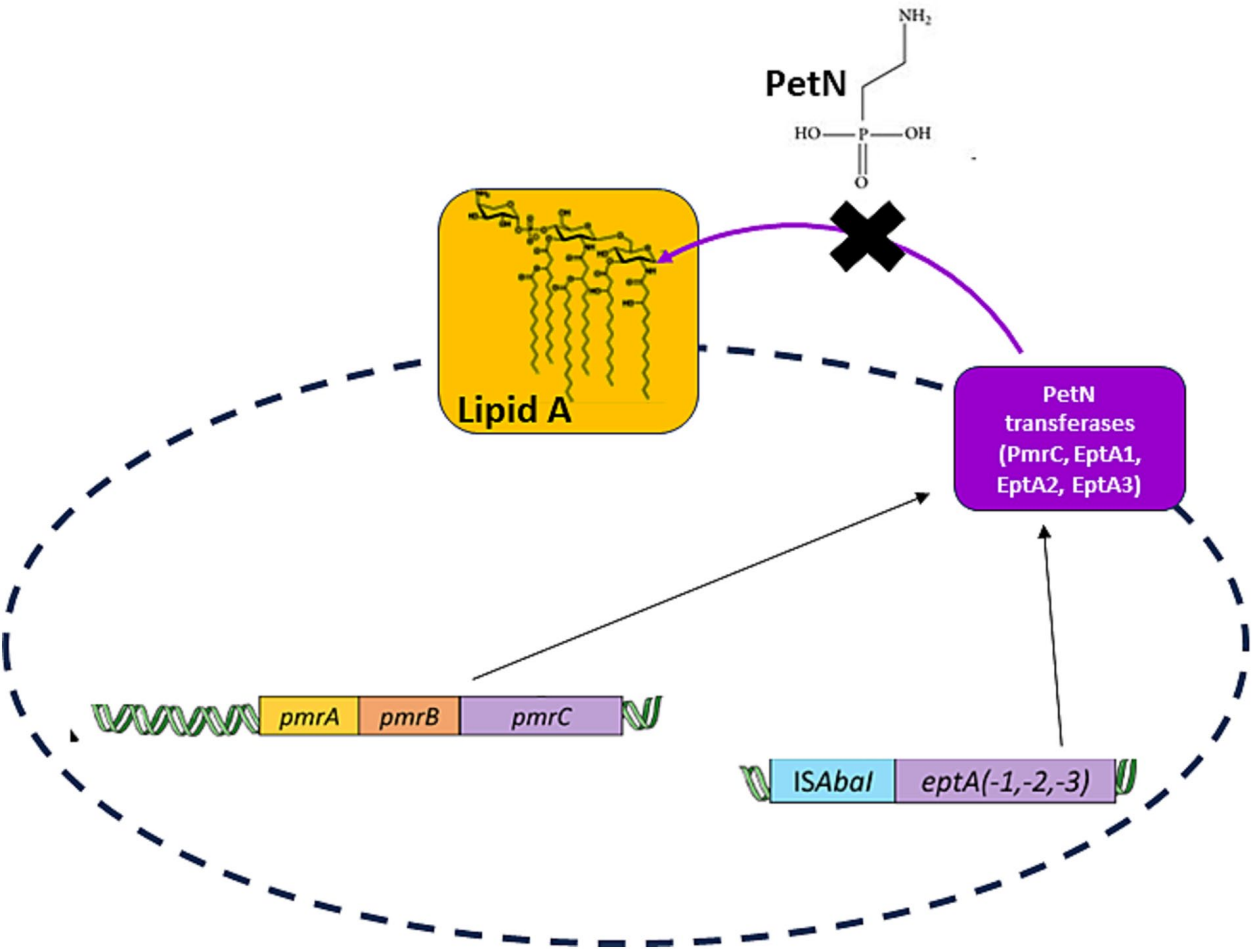
A scheme of the desired strategy, the inhibition of all PetN transferases independent of their transcriptional regulator. PmrC transcription is regulated by PmrA, whereas transcription of homologous PetN transferases (EptA, EptA-1, and EptA-2) is regulated by a different promoter, ISAbaI, at different genome location (Trebosc et al., 2019).

In *A. baumannii* clinical isolates, colistin resistance is mainly associated with alterations in the pmrCAB operon. The *pmrC* gene encodes for a PetN transferase, whereas pmrA and pmrB encode for the expression regulating a two-component system (Trebosc et al., 2019). Since PmrA is the transcriptional regulator that triggers PmrC overexpression, it has been considered a drug target, as its inhibition may potentially block PmrC overexpression and switch off colistin resistance (Harris et al., 2014). Additionally, the crystal structure of the receiver domain of PmrA has recently been determined, confirming that PmrA activates transcription by binding to a conserved DNA motif within the promoters of two operons, *pmrC* and *naxD* genes (Ouyang et al., 2024). However, it was previously shown that in the absence of PmrA-mediated expression of PmrC, overexpression of alternative, highly similar PetN transferases—EptA, EptA1, and EptA2—occurs. These enzymes are all capable of conferring colistin resistance in *A. baumannii* clinical isolates (Trebosc et al., 2019). Consistently, the expression of homolog EptA transferases is regulated by a different promoter, ISAbaI, at a different genome location (Trebosc et al., 2019; Figure 1).

In this study, we focused on investigating structural features of the PetN transferase PmrC by combining computational and biophysical studies. Using molecular modelling and virtual screening approaches, we opted to identify ligands of PmrC, which could potentially block its biological action. We identified a potential ligand, which we experimentally proved to bind recombinant PmrC with μM affinity. This ligand can drastically reduce colistin MIC in *A. baumannii*-resistant strains. Importantly, we identified strong residue conservation of the ligand binding pocket in all PetN transferase homologues. This study highlights PetN transferases as important drug targets and provides a robust prerequisite for designing a universal strategy against all PetN transferases to oppose colistin resistance in *A. baumannii*.

## 2 Experimental methods

### 2.1 Protein expression and purification

The gene sequence encoding the membrane protein PmrC was cloned into the pETM13 expression vector. The recombinant vector was transformed in ClearColi BL21(DE3) - Biosearch Technologies competent cells to overpopulate the protein. The ClearColi cells, which have a genetically modified lipopolysaccharide (LPS), were grown in 2.5 L of Luria Bertani (LB) medium (Applichem) medium containing 50-μg ml^−1^ Kanamycin at 37°C to an optical density (OD) 600 of ∼0.6–0.7. Isopropyl β-d-1-thiogalactopyranoside (IPTG) was added to a final concentration of 0.8 mM, and the culture was incubated overnight at 25°C. All purification procedures were performed at 4°C. The harvested cell pellet derived from 2.5 L of culture was resuspended in lysis buffer (200-mM NaCl, 5% (v/v), glycerol, and 50-mM 2-[4-(2-hydroxyethyl)piperazin-1-yl]ethanesulfonic acid [Hepes], pH 7.4) containing complete protease inhibitor cocktail (Roche Diagnostics, Mannheim, Germany) and was lysed using a digitally controlled high-pressure homogeniser LM20 Microfluidizer® (Microfluidics^TM^). The lysate was centrifuged at 10,000 rpm for 10 min, and the supernatant, containing inside-out membrane vesicles (ISOVs), was centrifugated at 23,000 rpm for 2 h. The resultant pellet was solubilised in a buffer containing 200-mM NaCl and 50-mM Hepes (pH 7.4), and 5% (v/v) of glycerol using a Potter–Elvehjem homogenizer. The solubilised suspension was incubated with stirring for 1 h in the presence of 1% n-dodecyl-β-d-maltopyranoside (DDM). The supernatant, enriched with 1 ml of Ni-NTA resin (Qiagen) per 1 mg of protein, was incubated with stirring for 1 h. It was subsequently loaded onto an Econo-Column (Bio-Rad Laboratories) (Bio-Rad) that had been pre-washed with 10 Column volumes of a buffer containing 10 mM imidazole and 0.1% DDM. The membrane protein PmrC with the His tag at the C-terminus was eluted in the same buffer containing 250 mM imidazole. The eluted protein was dialyzed overnight in the presence of a buffer containing 200 mM NaCl, 20 mM Hepes (pH 7.4), glycerol 5, and 0.03% DDM. The protein was concentrated using a 100-kDa cutoff concentrator and injected in a Superdex S200 (Cytiva) 10/300 size exclusion column (GE Healthcare) equilibrated with the dialysis buffer. The fraction corresponding to PmrC was concentrated to 1.3 mg ml^−1^ for further analysis.

### 2.2 CD spectroscopy

Circular dichroism (CD) spectra were measured using a Jasco J-810 spectropolarimeter with a Peltier temperature control system (Model PTC-423-S, Jasco Europe, Cremella (LC), Italy). Far-ultraviolet (UV) measurements at a protein concentration of 0.2 mg/ml were carried out at 20°C in 20-mM phosphate buffer pH 7.4 using a 0.1-cm optical path length cell and varying the wavelength from 260 to 198 nm at 20 nm min^−1^ scan rate. The molar ellipticity per mean residue, [Ѳ] in deg.·cm^2^·dmol^−1^, was calculated from the following equation: [Ѳ] = [Ѳ]obs × MRW × (10 × l × C)^−1^, where [Ѳ]obs is the ellipticity measured in degrees, MRW is the mean residue molecular mass, C is the protein concentration in g/L, and l is the optical path length of the cell in cm. Mean residue molecular mass is calculated from MRW = M/(N − 1), where M is the protein’s molecular mass (in Da) and N is the number of amino acid residues. This value for PmrC is 113,12. CD melting curve was registered as a function of temperature (range: 20–90°C) at their maximum Cotton effect wavelengths with a scan rate of 1°C/min.

### 2.3 Molecular modelling

The three-dimensional (3D) structure of the whole PmrC protein was obtained using homology modelling and the MODELLER 9.22 program^1^ (Webb and Sali, 2021) starting from the crystal structure of integral membrane protein lipooligosaccharide phosphoethanolamine transferase A (EptA) from *Neisseria meningitidis* (PDB code 5fgn, sequence identity 36.38%) as a template (Anandan et al., 2017). In parallel, modelling was performed using the most recently released AlphaFold3.0 modelling server,^2^ which generated five models, ranked by AlphaFold3.0 ranking score (Abramson et al., 2024).

### 2.4 Virtual screening

The selection of potential PmrC binder candidates was performed by high-throughput docking of the LifeChemicals commercial molecular database on the PmrC 3D model. The protein 3D structures were prepared using the Protein Preparation Wizard tool of the Schrödinger Suite 2015–4 (Schrödinger, LLC, New York, NY, USA). The binding site occupied by DODECYL-BETA-D-MALTOSIDE (Anatrace) in the 5fgn PDB structure was used as a guide to define the PmrC pocket to target after superimposing the two proteins. The grid box was built using DODECYL-BETA-D-MALTOSIDE as the centre and as a dimensional reference. The LifeChemicals database,^3^ downloaded as smi files from ZINC^4^ (Irwin et al., 2012), was treated with the Schrödinger Suite 2015–4 (Schrödinger, LLC, New York, NY, USA) to generate all the corresponding 3D structures at pH 7.4 ± 1.0 using the LigPrep tool (part of Schrödinger – official website^5^, Epik LigPrep tool (part of Schrödinger – official website (See footnote 5)), and OPLS2005 LigPrep tool (part of Schrödinger – official website (See footnote 5)) as the force field. A first docking calculation of all the compounds against the protein model was performed using Glide LigPrep tool (part of Schrödinger – official website (See footnote 5)) (Friesner et al., 2006) in standard precision (SP). Based on the docking scores, 2000 hits were selected. These selected molecules were then docked in extra precision (XP) mode and visually inspected to choose finally 44 molecules The 44 molecules were then subjected to a standard protocol of induced fit docking (IFD) using Glide. Finally, 26 molecules were chosen for purchase from LifeChemicals.

### 2.5 Microscale thermophoresis binding studies

The thermophoretic measurements were conducted using the Monolith NT.115 device (NanoTemper Technologies, Munich, Germany) equipped with a red detection channel. For MST analysis, recombinant PmrC was labeled with the fluorescent dye RED-NHS 2nd Generation (NanoTemper Technologies) provided by Protein Labeling Kit RED-NHS (NanoTemper Technologies) (NanoTemper Technologies). This fluorescent dye carries a reactive NHS-ester group that reacts with primary amines to form a covalent bond. Briefly, 90 μl of a 10-μM PmrC solution in labeling buffer (130-mM NaHCO_3_, 50-mM NaCl, and pH 8.2) was mixed with 10 μl of 300-μM dye RED-NHS Second Generation and incubated in the dark for 30 min at room temperature. Following labeling, the protein bound to the fluorophore was separated from any unreacted excess fluorophore using a specific column provided in the NanoTemper kit. The concentration of PmrC after labeling with the fluorescent dye was determined using an ultraviolet–visible (UV–Vis) spectrophotometer, with a labeling efficiency of approximately 25%. The MST experiment was conducted in a buffer containing phosphate buffer solution (PBS) at pH 7.4 and 0.03% DDM. Approximately 10 μl of labeled PmrC at 13 nM was mixed with 10-μl of 16 serial dilutions of compound F3205-0035. The final concentration of labeled PmrC was 6.5 nM in all samples, while the concentration of F3205-0035 ranged from 3.6 mM to 0.1 μM. Samples were then loaded into 16 premium-coated capillaries provided by NanoTemper Technologies, and fluorescence was recorded for 20 s using 100% laser power and 40% MST power. The instrument temperature was maintained at 25°C throughout all measurements. After recording the MST time traces, the data were analysed. The dissociation constant (Kd) value was calculated based on the ligand concentration-dependent changes in the normalised fluorescence (F_norm_) of labeled PmrC after 14 s of thermophoresis. The assay was conducted in duplicate, and the reported values were generated using MO Affinity Analysis software provided by NanoTemper Technologies.

### 2.6 Bacterial strains

*A. baumannii* Ab4451 (Durante-Mangoni et al., 2015) and Ab60537 (Stabile et al., 2023) clinical isolates belonging to a collection previously established at the Department of Molecular Medicine and Medical Biotechnology, University of Naples Federico II, were routinely grown on Luria–Bertani agar plates (Del-tek) at 37°C under aerobic conditions. Stock cultures were stored in 10% glycerol and maintained at −80°C until use. In biological assays, the strain was grown in fresh cation-adjusted Mueller–Hinton broth (CA-MHB) (Sigma-Aldrich). A stock solution of colistin sulfate salt (Sigma–Aldrich) at 50 mg/ml concentration was obtained by dissolving the powder in H_2_O.

### 2.7 Antibiotic susceptibility testing

MIC values for s-Phen and colistin against planktonic bacteria were assessed using a manual microdilution method in accordance with the guidelines suggested by the European Committee for Antimicrobial Susceptibility Testing (Esposito et al., 2019). Briefly, overnight grown bacterial culture was diluted in saline to 0.5 McFarland turbidity using a BD PhoenixSpec™ nephelometer (Becton Dickinson, Franklin Lakes, NJ, USA), followed by a 1:100 further dilution in CA-MHB to a final concentration of approximately 5 × 10^6^ colony forming unit (CFU)/ml. The bacterial culture (100 μl) was then added to a 96-well microtiter plate containing 100 μl of 2-fold serial dilutions in CA-MHB of s-Phen or colistin and incubated at 37°C. Each plate contained wells with no compounds as a positive growth control and serially diluted colistin or s-Phen in CA-MHB as a negative control. In each well DMSO concentrations were nontoxic for bacterial growth. After 18 h of incubation, the absorbance of bacterial culture at 595 nm was determined using a microplate reader (Bio-Rad Laboratories S.r.l., Hercules, CA, USA). The MIC values for the tested compounds were determined as the lowest concentration that inhibited bacterial growth.

Checkboard assays were carried out to test the MIC of the combination of colistin and s-Phen (De Gregorio et al., 2020). Briefly, 100 μl of the bacterial culture at a concentration of 5 × 10^6^ CFU/ml was added to a 96-well microtiter plate, where each well contained 100 μl of colistin (0–512 μg/ml), s-Phen (0, 200, and 400 μM), or colistin plus s-Phen (at the same final concentration to the previously mentioned one). The plates were incubated at 37°C for 18 h, and MIC values were measured by recording OD_595_ of each well.

### 2.8 RNA experiments

*A. baumannii* cultures were grown in LB broth at 37°C with continuous shaking until reaching an OD at 600 nm (OD600) of 0.4. Cells were then harvested from 2 ml of culture by centrifugation, and total RNA extraction was carried out according to the previously reported method (Migliaccio et al., 2021). Subsequently, the extracted RNA underwent treatment with RNase-free DNase (Invitrogen), and quantification was performed using a NanoDrop 1,000 spectrophotometer (Thermo Scientific, Winsford, UK).

For reverse transcription polymerase chain reaction (RT-PCR) analysis, 1,000 ng of total RNA was reverse transcribed using the QuantiTect Reverse Transcription Kit (Qiagen), according to the manufacturer’s instructions. The quantitative RT-PCR was performed using the SYBR Green master mix (Thermo Fisher Scientific) as previously described (Vollaro et al., 2020) with the ABI 7500 FAST instrument (Thermo Fisher Scientific) (Applied Biosystems). The primers used for PCR are listed in Supplementary Table S4. The 16S rRNA gene was used as an internal control for relative gene expression quantification based on the mean of three independent experiments. Negative controls without reverse transcriptase were included to detect possible DNA contamination. Fold changes in gene expression were determined using the 2^−ΔΔCt^ method (Livak and Schmittgen, 2001).

### 2.9 Haemolysis assay

The haemolytic activity of s-Phen was evaluated as previously described (Esposito et al., 2019). Briefly, 190 μl of a red blood cell (RBC) suspension diluted 50-fold in PBS was incubated with 10 μl of s-Phen in a 96-well plate. The negative control was PBS, and the positive control was PBS with Triton X-100 (Sigma-Aldrich) (1%). The plate was incubated at 37°C for 1 h, and then the plate was centrifuged at 500 rpm for 5 min to pellet the intact RBCs. After centrifugation, 100 μl of the supernatant from each well was transferred to a new 96-well plate. The absorbance of the supernatant was measured at 450 nm using a microplate reader. The haemolysis rate was calculated by using the following formula: 100 × (A_sample_ − APBS)/(A_Triton X-100_ − APBS), where A_sample_ is the experimental absorbance of s-Phen, APBS is the control absorbance of untreated erythrocytes, and A_Triton X-100_ is the absorbance of 1% Triton X-100-lysed cells. Statistical analyses were performed using GraphPad Prism 8 software (GraphPad, San Diego, CA, USA). All data points are expressed as the mean ± standard deviation (SD) of three independent experiments, each performed in triplicate. The statistical significance of the differences was determined using one-way analysis of variance (ANOVA), followed by Dunnett’s multiple comparison test to compare the groups.

## 3 Results and discussion

### 3.1 Biophysical characterisation of PmrC

**Figure 2:**
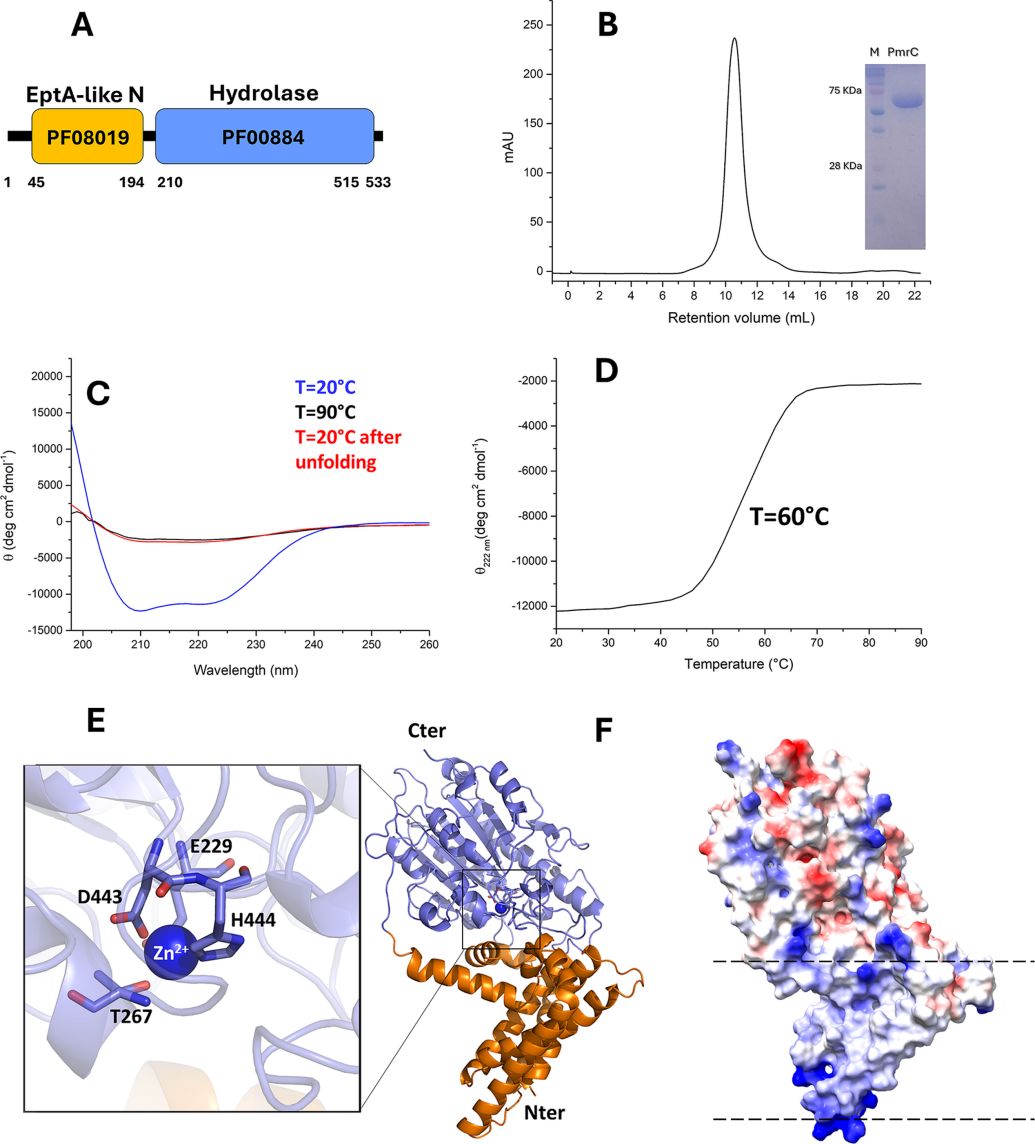
PmrC is a stable protein with α-β fold. (A) Domain borders of PmrC, computed using the Pfam database (Mistry et al., 2021). Pfam domain numbers are reported for the N- (orange box) and C-terminal (light blue box) domains. (B) Gel filtration chromatogram of PmrC. The inset shows the SDS-PAGE gel of purified PmrC; Markers lane is denominated as “M.” (C) CD spectra of PmrC at 20, 90, and 20°C after melting (D) Thermal unfolding profile of PmrC. (E) Cartoon representation of PmrC model structure, obtained by homology modelling and the software MODELLER, using the structure of EptA from *Neisseria meningitis*, (PDB code 5fgn; seqid 37%) as a template. The N- and C-terminal domains are drawn in slate blue and orange, respectively. The inset shows a zoom of the catalytic centre. (F) The electrostatic potential surface of PmrC, computed with ChimeraX (Meng et al., 2023). Dotted lines highlight aligned positive charges, likely interacting with phosphate groups of the membrane.

PmrC is long been recognised as a major responsible for colistin resistance in *A. baumannii* (Gogry et al., 2021; Hamel et al., 2021; Nurtop et al., 2019; Seleim et al., 2022; Trebosc et al., 2019). Despite its importance, a biophysical characterisation is still unavailable, likely due to its molecular complexity. PmrC is a 533-residue membrane protein (Figure 2A). Thus, we expressed and purified it in high yields with the detergent dodecyl-β-d-maltoside (DDM; Figure 2B). Far-UV CD spectroscopy spectrum of PmrC is typical of well-structured folds with high α-helical content, with typical minima at 208 and 222 nm (Figure 2C). Thermal unfolding curves were recorded by following the CD signal at 222 nm as a function of temperature, using a 1°C/min heating rate to investigate the heat-induced changes in the protein secondary structure. The thermal unfolding curve exhibits the characteristic sigmoidal profile expected of two-state systems, with a melting temperature T_m_ = 60°C (Figure 2D). Consistent with the complexity of the molecule, its folding is fully irreversible (Figure 2E).

We homology modelled PmrC using MODELLER (Webb and Sali, 2021) and the structure of EptA from *N. meningitis*, sharing 37% sequence identity with PmrC, as a template (Anandan et al., 2017). This model was fully superposable with that computed using AlphaFold3.0 (Abramson et al., 2024), with a root mean square deviation on Cα atoms of 2.0 Å. The PmrC structure model is formed by two distinct domains with completely different features. The N-terminal domain (residues 1–194, orange) is mostly hydrophobic and contains 5 transmembrane helices (α1–α5; Figure 2E). This domain is predicted to be fully immersed in the membrane double layer. Consistently, the protein electrostatic potential map of the protein shows patches of positively charged residues at the two extremities of the N-terminal domain (Figure 2F), likely mediating charge interactions with the phosphate groups of the membrane phospholipids. The C-terminal domain of PmrC (residues 200–533) is an alpha-beta domain typical of the hydrolase-type fold (Figure 2E). Structural alignment using DALI (Holm et al., 2023) shows consistently similar domains in sulfatases and phosphatases (Supplementary Table S1). Similar to EptA from *N. meningitis* (Anandan et al., 2017) and MCR-1 of *E. coli* (Suardíaz et al., 2021), PmrC C-terminal domain contains a Zn^2+^ binding site, composed by Glu229, Thr267, Asp443, and His444 (Figure 2E).

### 3.2 Identification of PmrC ligands by virtual screening

The best characterised PetN transferase catalytic mechanism is that of MCR-1 of *E. coli*, which shares 37% identity with PmrC (Suardíaz et al., 2021). MCR-1 is an integral, Zn^2+^-dependent inner-membrane protein. The zinc site is tetrahedrally coordinated in MCR-1 residues Glu246, Asp465, His466, and Thr285, corresponding to Glu229, Asp443, His444, and Thr267 in PmrC, respectively (Figure 2E). In MCR-1, the reaction path for PetN transfer from the bacterial lipid membrane to the protein involves a nucleophilic attack of Thr285 (267 in PmrC) on the phosphate centre of the phospholipid, concerted with the activation of Thr285 by Glu246 and a proton transfer from His395 to the dephosphorylated lipidic leaving group (Suardíaz et al., 2021). To inactivate this reaction mechanism, a virtual screening campaign based on high throughput docking (HTPD) was conducted on the modelled structure of PmrC. Docking of the LifeChemicals (See footnote 2) commercial database was performed in Standard Precision (SP) mode using Glide (Schrödinger, LLC, New York, NY, 2015). The top 200 compounds based on docking scores were saved and redocked in extra precision (XP) mode. After visual inspection, 44 compounds were selected and subjected to Induced Fit Docking (IFD) with Glide. Visual inspection criteria were steric complementarity of compounds with the binding site, hydrogen bonds, and hydrophobic contacts (Fischer et al., 2021). Finally, after IFD, 26 compounds were chosen for purchase and subsequent testing (Supplementary Table S2).

### 3.3 s-Phen ligand binds to a hydrophobic pocket of PmrC

**Figure 3:**
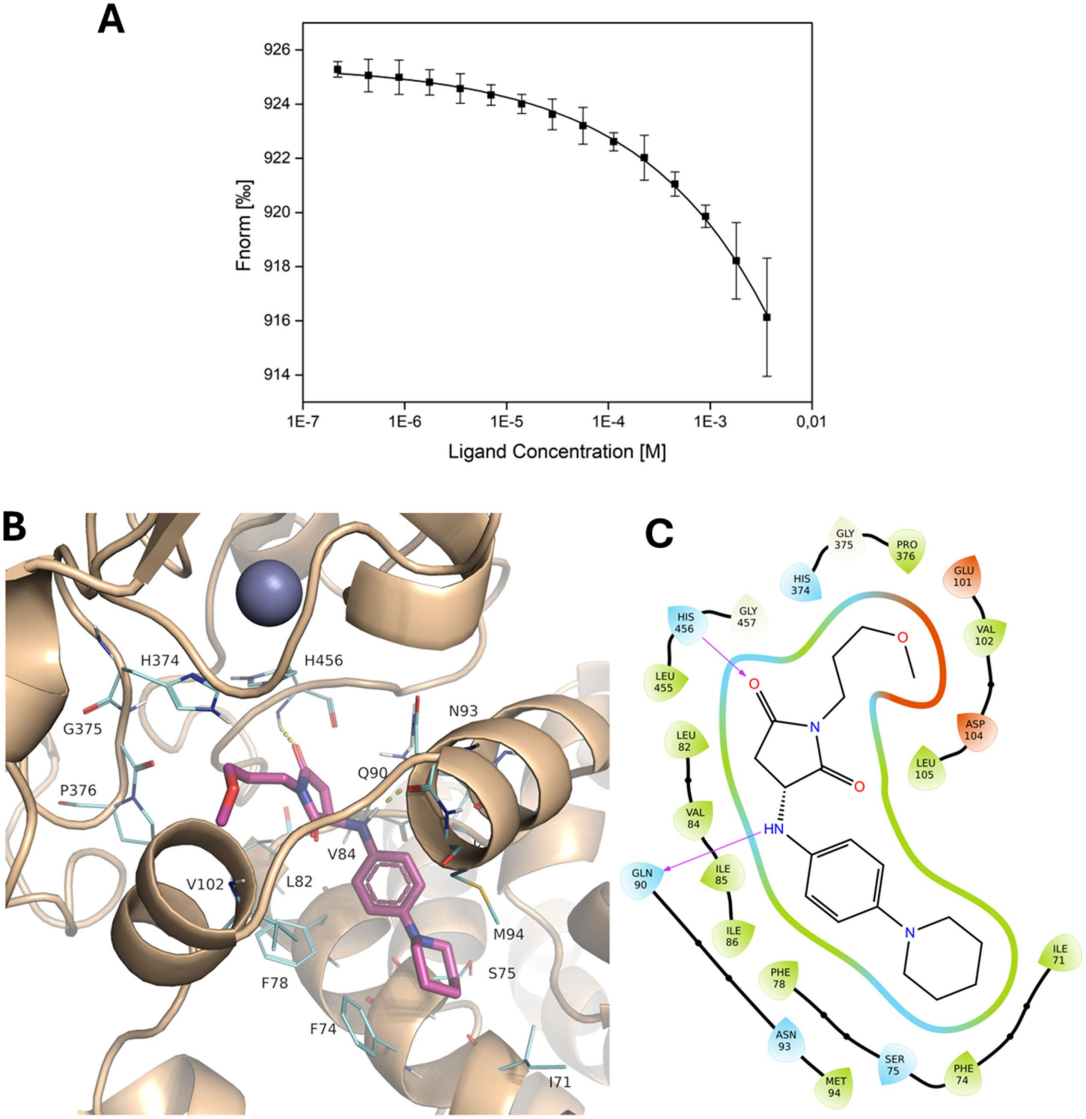
Binding of s-Phen in the binding pocket of PmrC. (A) Binding isotherm for MST signal versus s-Phen concentrations. (B) Localisation of s-Phen in PmrC binding pocket. S-Phen is drawn in stick representation, and PmrC is in the cartoon. s-Phen contacting residues are drawn in line. (C) 2D protein-ligand interaction diagram. The hydrophobic cavity is represented in green, polar residues are shown in cyan, and charged residues are depicted in red. Hydrogen bonds are indicated by violet arrows.

The selected compounds were solubilised for experimental assessment of their ability to interact with PmrC. The majority of soluble molecules under conditions compatible with binding experiments were F3320-0359, F3205-0035, F6761-7637, F6523-3945, and F6231-0947 (Supplementary Table S2), which were tested for their binding to PmrC using MST. We observed that, among the tested compounds, increasing concentrations of F3205-0035 clearly affect the thermophoretic motion of PmrC (Figure 3A; Supplementary Figure 1). F3205-0035 is a 1-(3-methoxypropyl)-3-{[4-(piperidin-1-yl)phenyl]amino}pyrrolidine-2,5-dione, and is here denominated as s-Phen. Although the low solubility of s-Phen did not allow us to reach saturation, we observed a clear dose–response curve, and the data fitting provided a *K*_d_ value in the high micromolar range (Figure 3A). Docking of s-Phen in the catalytic site cleft of PmrC shows that s-Phen fills a deep pocket located at the interface between the N-and C-terminal domains (Figures 3B,C). Several hydrophobic interactions characterise the binding to residues of the N-terminal domain, including Phe74, Phe78, Leu82, Val84, Ile85, Ile86, and with Pro376 and Leu455 of the C-terminal domain (Figure 3C). In addition, hydrogen bonding interactions occur with the main chain atoms of Gln90 (O) and His456 (N; Figure 3C). Of note, s-Phen does not contact any of the residues hitherto known to contribute to PmrC catalysis, as the majority of its interactions with the protein occur with the N-terminal domain. However, it occupies the same hydrophobic cavity of DDM (Supplementary Figure 2), which mimics acyl chains of lipid A, as previously suggested for the homolog EptA from *N. meningitis*(Anandan et al., 2017). Therefore, s-Phen likely acts by blocking the cavity, which accommodates the hydrophobic acyl chains of lipid A (Supplementary Figure 2).

### 3.4 s-Phen hampers colistin resistance in *A. baumannii* clinical isolates

**Figure 4:**
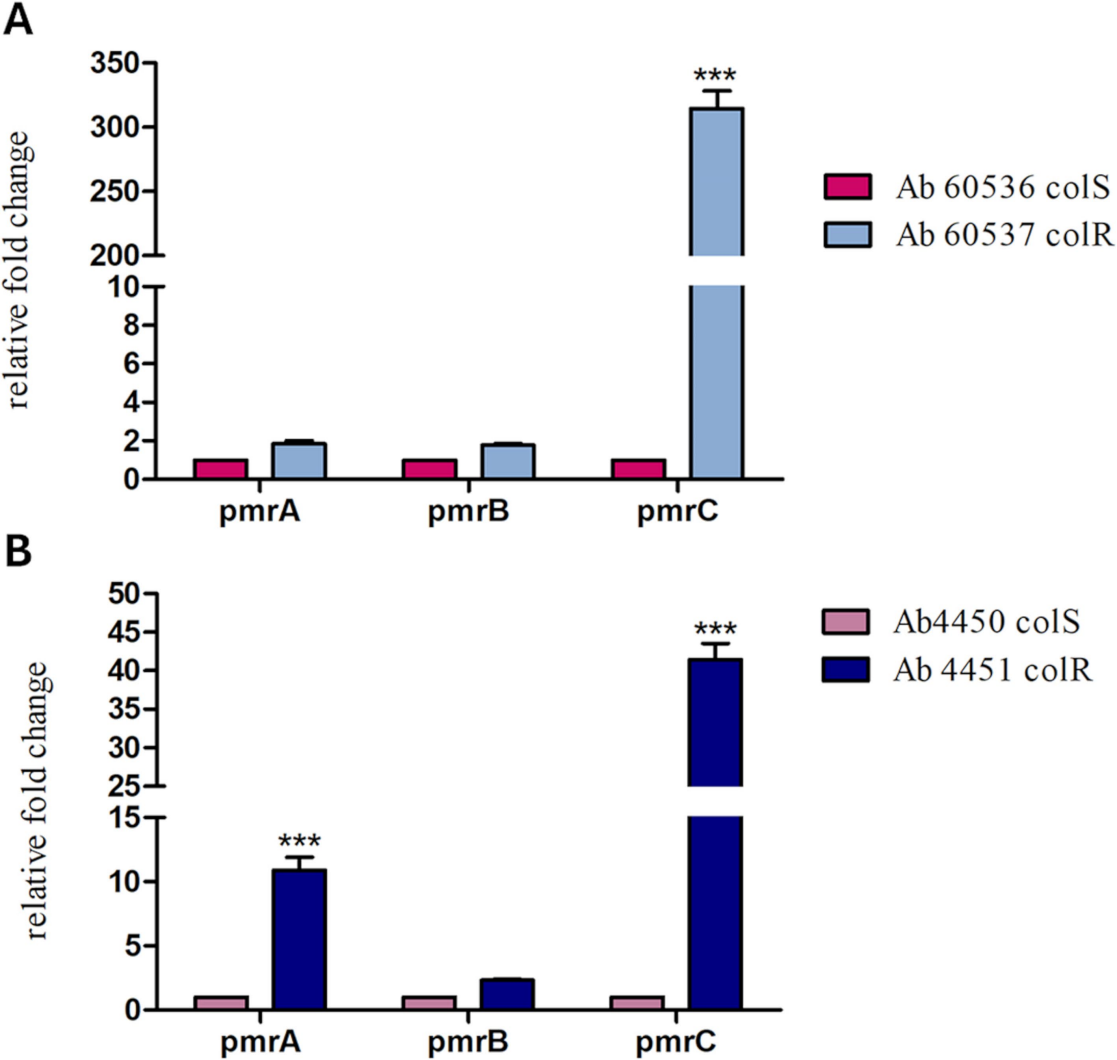
The relative expression of *pmrA*, *pmrB*, and *pmrC* in two colistin-susceptible/colistin-resistant pairs of *Acinetobacter baumannii* clinical isolates, Ab60536/Ab60537 (A) and Ab4450/Ab4451 (B), was determined by qRT-PCR. The number of transcripts of colistin-resistant isolates was related to that of colistin-susceptible isolates after being normalised to the expression of the housekeeping gene *rpoB* and the susceptible isolate, set to 1. The results are represented as mean ± standard error of the mean. Statistical analysis was performed using the one-way analysis of variance (ANOVA). ***p < 0.001.

**Figure 5:**
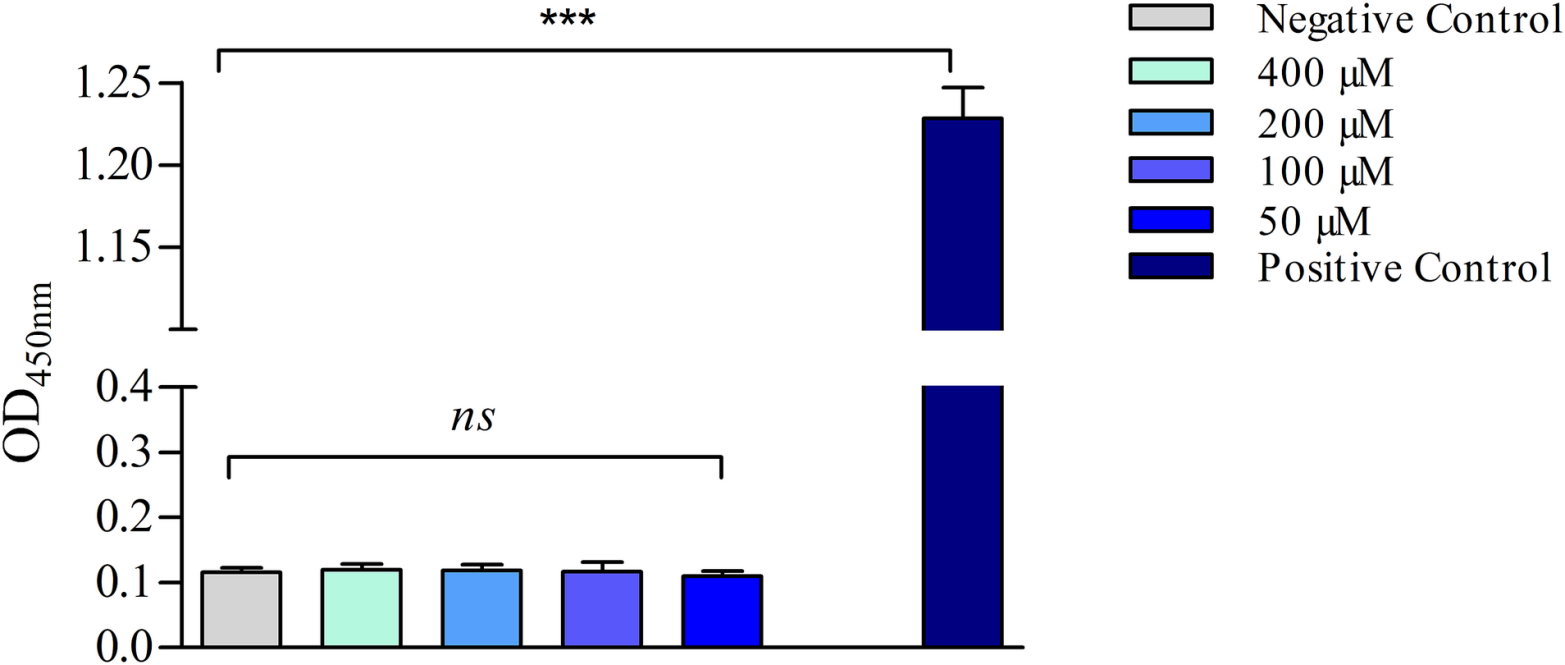
Haemolysis assay. The samples were incubated for 60 min at 37°C with either PBS (negative control), 1% (v/v) Triton X-100 (positive control), or different concentrations of s-Phen. The data are expressed as the mean ± standard deviation (SD) of three replicates. ***p < 0.001, significance was analyzed using the one-way analysis of variance (ANOVA); ns, not statistically significant.

The efficacy of s-Phen in contrasting colistin resistance was tested in the two colistin-resistant clinical isolates, Ab60537 (Stabile et al., 2023) and Ab4451 (Durante-Mangoni et al., 2015). The colistin-resistant strains Ab4451 and Ab60537 present amino acid substitutions in the PmrB sensor kinase of the PmrAB TCS. It has been shown that mutations in the PmrAB lead to the overexpression of pmrC, conferring resistance to colistin (Arroyo et al., 2011; Trebosc et al., 2019). First, we performed quantitative reverse transcription-PCR (qRT-PCR) to investigate *pmrABC* gene expression in two colistin-susceptible/colistin-resistant pairs of *A. baumannii* clinical isolates (Ab60536/Ab60537 and Ab4450/Ab4451). As shown in Figure 4, the colistin-resistant isolates Ab60537 and Ab4451 had only slightly increased expression of *pmrA* (1.84- and 10.9-fold, respectively) and *pmrB* (1.77- and 2.33-fold, respectively) compared to their colistin-susceptible counterparts Ab4450 and Ab60536, whereas they showed a marked increase in *pmrC* expression (314.5- and 41.45-fold, respectively). This suggested that colistin resistance in the isolates Ab60537 and Ab4451 is due to PmrC overexpression. After this preliminary check, we tested the five compounds F3320-0359, F6761-7637, F6523-3945, F6231-0947, and s-Phen for antimicrobial activity. Consistent with binding studies, we observed that all compounds do not act as antimicrobials by themselves, as in all cases, MIC values were > 150 μg/ml. However, when added to colistin, s-Phen was able to drastically reduce the MIC value of colistin in both Ab60537 and Ab4451-resistant isolates (Table 1). In the presence of s-Phen at its maximum concentration of 138 μg/ml, colistin MIC decreased 16-fold (from 128 to 8 μg/ml) for Ab60537 and 64-fold (256–4 μg/ml) for Ab4451 (Table 1). We also measure the cytotoxicity of s-Phen by haemolysis assay against red blood cells to assess its safety profile. The results indicate that s-Phen is not toxic, as evidenced by haemolysis levels similar to those of the negative control (Figure 5). Indeed, s-Phen does not cause measurable haemolysis at any of the concentrations tested.

**Table 1:** MIC values of s-Phen, colistin, and s-Phen/colistin combinations against colistin-resistant clinical isolates.

Bacterial colistin-resistant strains	s-Phen MIC (μg/ml)	Colistin MIC (μg/ml)	Colistin MIC (μg/ml) + 69 μg/ml s-Phen	Colistin MIC (μg/ml) + 138 μg/ml s-Phen
Ab60537	>150	128	64	8
Ab4451	>150	256	128	4

## 4 Discussion

**Figure 6:**
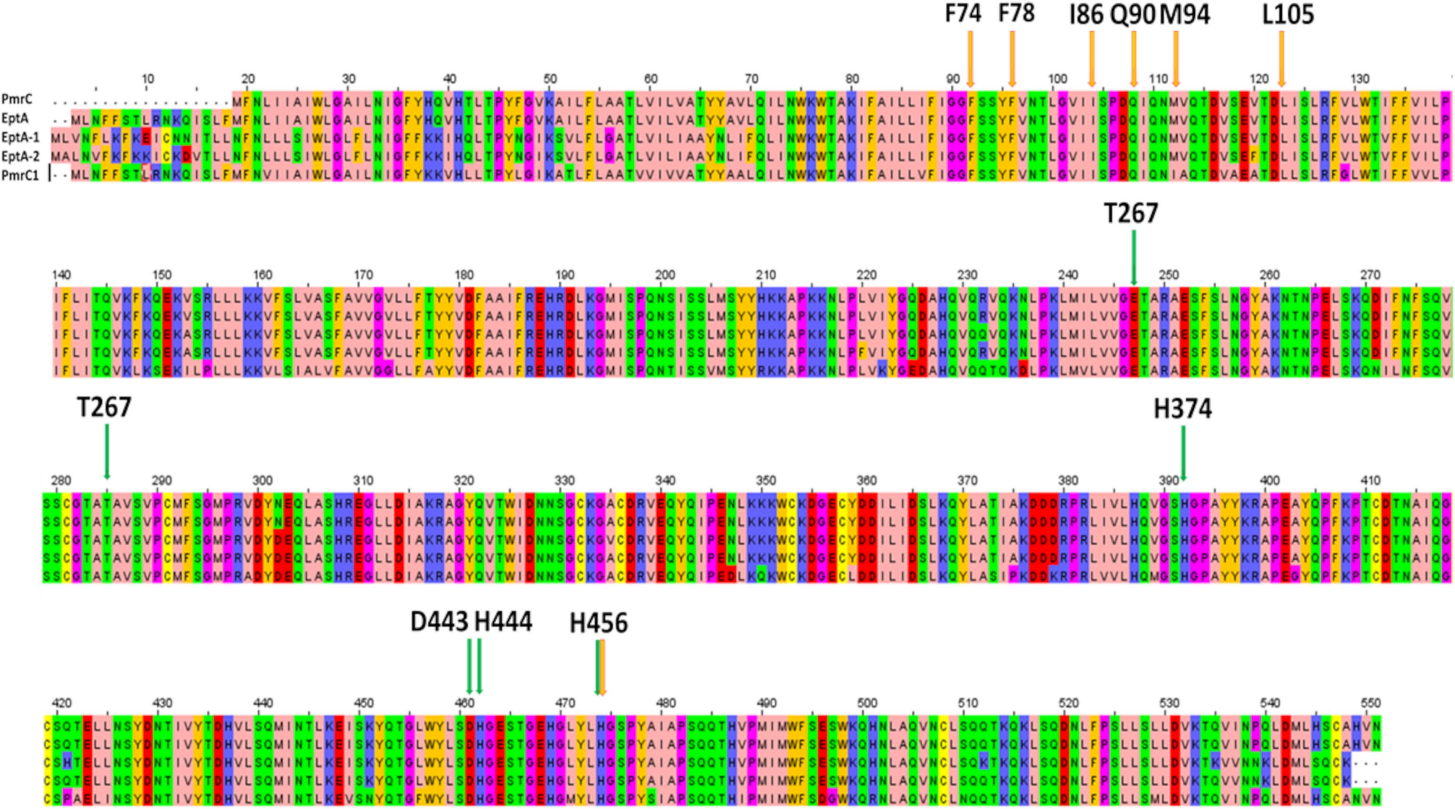
Sequence alignment of clinically isolated PetN transferases, performed with Clustalw (https://www.genome.jp/tools-bin/clustalw) and visualised using Jal View (https://www.jalview.org/). Important residues for catalysis are highlighted by green arrows, whereas residues involved in s-Phen binding are indicated by the orange arrows.

Colistin is one of the few therapeutic options for treating multiresistant *A. baumannii* infections. However, colistin resistance has rapidly emerged in patients due to antibiotic pressure (Bostanghadiri et al., 2024; Cafiso et al., 2018). The spread of resistance correlates with the actual use of colistin in clinical practice. In Western Europe, an increase in resistance has been recorded from 11% in 2019 to 31% in 2020, likely due to the widespread use of antibiotics during the severe acute respiratory syndrome coronavirus 2 (SARS-CoV-2) pandemic. The resistance level decreased by approximately 20% in 2021 after the SARS-CoV-2 outbreak (Bostanghadiri et al., 2024). Lower levels of resistance exist in countries that make poor use of colistin in clinical practice, particularly in the veterinary field (Bostanghadiri et al., 2024). Given the importance of colistin in cases of resistance to all other antibiotics, the emergence of colistin resistance is a threat to patient survival. It requires increased attention from the scientific community (Novović and Jovčić, 2023). Active surveillance of multidrug-resistant organisms has, over the years, provided an opportunity to study the evolution of colistin resistance in isolates of *A. baumannii* from patients undergoing antibiotic therapy. Indeed, several PmrC homologues have been identified in clinical strains, including PmrC1, EptA, EptA-1, and EptA-2 (Lesho et al., 2013; Trebosc et al., 2019; Supplementary Table S3). Compared to colistin-susceptible isolates, colistin-resistant isolates were found to display significantly enhanced expression of pmrC1A1B, eptA-1, and eptA-2 (Lesho et al., 2013). Interestingly, although these clinically relevant PmrC homologues are almost identical, genes encoding for EptA-1 and EptA-2 were found to be distant from the operon encoding for PmrC (Lesho et al., 2013). Sequence identity between PmrC and its homologues ranges between 81 and 97% (Supplementary Table S3), with PmrC being the shortest and missing the N-terminal 18 residues (Figure 6). Full conservation characterises residues which that are putatively involved in catalysis, as well as those that interact with the identified compound (Figure 6). These considerations prompted us to search for inhibitors of this family of proteins as adjuvants of colistin with a strong potential to inactivate all PetN transferases regardless of the location of genes encoding for them and of their operons. First, we recombinantly produced and biophysically characterised PmrC, showing that it is a well-folded and stable protein, with T_m_ = 60°C. Consistent with our CD analysis, showing typical features of α-β protein structures, homology modelling of PmrC highlights similar structural features compared to its homologue from *N. meningitis* (Anandan et al., 2017). Using this model, we adopted a virtual screening approach to identify a set of potential binders. Thermophoresis assays showed that one compound, here s-Phen, was able to bind PmrC with μM binding affinity. Docking of s-Phen in the catalytic site cleft suggested that s-Phen acts by blocking the cavity, which accommodates the hydrophobic acyl chains of lipid A. Indeed, our analysis identified several amino acids belonging to a deep hydrophobic cavity of the PmrC N terminal domain, which plays a crucial role in the inhibition of the PmrC mechanism, including Phe74, Phe78, Leu82, Val84, Ile85, and Ile86. Consistently, microbiological assays on *A. baumannii* clinical isolates proved that the binding to PmrC is responsible for a drastic reduction in colistin resistance. Although further work still needs to be done to increase the efficacy of the s-Phen compound as an inhibitor of PmrC and homologous EptAs, our results identify PmrC inactivation as a valid molecular mechanism for the design of synergistic molecules to combat colistin-resistant *A. baumannii* strains. In conclusion, we prove the success of structure-based virtual screening approaches to identify novel antibiotic adjuvants. Also, the full conservation in all PmrC homologues of amino-acid residues interacting with s-Phen (Figure 6) predicts the full usage of this class of molecules as adjuvants of colistin, regardless of the operon involved in resistance. Antibiotic adjuvants offer a promising solution to combat MDR pathogens. By addressing the challenges posed by the specific antibiotic resistance mechanism, structure-based designed adjuvants can revolutionise the treatment of resistant infections.

## Data Availability

The original contributions presented in the study are included in the article/Supplementary material, further inquiries can be directed to the corresponding authors.

## Footnotes

^1^ https://salilab.org/modeller/

^2^ https://www.alphafoldserver.com

^3^ http://www.lifechemicals.com

^4^ https://zinc.docking.org/

^5^ https://www.schrodinger.com/

## References

[ref1] Abramson J. Adler J. Dunger J. Evans R. Green T. Pritzel A.. (2024). Accurate structure prediction of biomolecular interactions with AlphaFold 3. Nature 630, 493–500. doi: 10.1038/s41586-024-07487-w, PMID: 38718835 PMC11168924

[ref2] Anandan A. Evans G. L. Condic-Jurkic K. O’Mara M. L. John C. M. Phillips N. J.. (2017). Structure of a lipid a phosphoethanolamine transferase suggests how conformational changes govern substrate binding. Proc. Natl. Acad. Sci. 114, 2218–2223. doi: 10.1073/pnas.1612927114, PMID: 28193899 PMC5338521

[ref3] Arroyo L. A. Herrera C. M. Fernandez L. Hankins J. V. Trent M. S. Hancock R. E. W. (2011). The pmrCAB operon mediates Polymyxin resistance in *Acinetobacter baumannii* ATCC 17978 and clinical isolates through phosphoethanolamine modification of lipid A. Antimicrob. Agents Chemother. 55, 3743–3751. doi: 10.1128/AAC.00256-11, PMID: 21646482 PMC3147623

[ref4] Bostanghadiri N. Narimisa N. Mirshekar M. Dadgar-Zankbar L. Taki E. Navidifar T.. (2024). Prevalence of colistin resistance in clinical isolates of *Acinetobacter baumannii*: a systematic review and meta-analysis. Antimicrob. Resist. Infect. Control 13:24. doi: 10.1186/s13756-024-01376-7 38419112 PMC10902961

[ref5] Cafiso V. Stracquadanio S. Lo Verde F. Gabriele G. Mezzatesta M. L. Caio C.. (2018). Colistin resistant *A. baumannii*: genomic and transcriptomic traits acquired under colistin therapy. Front. Microbiol. 9:3195. doi: 10.3389/fmicb.2018.03195, PMID: 30666237 PMC6330354

[ref6] De Gregorio E. Esposito A. Vollaro A. De Fenza M. D’Alonzo D. Migliaccio A.. (2020). N-nonyloxypentyl-l-deoxynojirimycin inhibits growth, biofilm formation and virulence factors expression of *Staphylococcus aureus*. Antibiotics (Basel) 9:362. doi: 10.3390/antibiotics9060362, PMID: 32604791 PMC7344813

[ref7] Durante-Mangoni E. Del Franco M. Andini R. Bernardo M. Giannouli M. Zarrilli R. (2015). Emergence of colistin resistance without loss of fitness and virulence after prolonged colistin administration in a patient with extensively drug-resistant *Acinetobacter baumannii*. Diagn. Microbiol. Infect. Dis. 82, 222–226. doi: 10.1016/j.diagmicrobio.2015.03.013, PMID: 25858028

[ref8] Esposito A. Gregorio E. D. Fenza M. D. D’Alonzo D. Satawani A. Guaragna A. (2019). Expeditious synthesis and preliminary antimicrobial activity of deflazacort and its precursors. RSC Adv. 9, 21519–21524. doi: 10.1039/C9RA03673C, PMID: 35521350 PMC9066171

[ref9] Fischer A. Smieško M. Sellner M. Lill M. A. (2021). Decision making in structure-based drug discovery: visual inspection of docking results. J. Med. Chem. 64, 2489–2500. doi: 10.1021/acs.jmedchem.0c02227, PMID: 33617246

[ref10] Friesner R. A. Murphy R. B. Repasky M. P. Frye L. L. Greenwood J. R. Halgren T. A.. (2006). Extra precision glide: docking and scoring incorporating a model of hydrophobic enclosure for protein−ligand complexes. J. Med. Chem. 49, 6177–6196. doi: 10.1021/jm051256o, PMID: 17034125

[ref11] Gogry F. A. Siddiqui M. T. Sultan I. Haq Q. M. R. (2021). Current update on intrinsic and acquired colistin resistance mechanisms in bacteria. Front. Med. 8:677720. doi: 10.3389/fmed.2021.677720, PMID: 34476235 PMC8406936

[ref12] Hamel M. Rolain J.-M. Baron S. A. (2021). The history of Colistin resistance mechanisms in bacteria: progress and challenges. Microorganisms 9:442. doi: 10.3390/microorganisms9020442, PMID: 33672663 PMC7924381

[ref13] Harris T. L. Worthington R. J. Hittle L. E. Zurawski D. V. Ernst R. K. Melander C. (2014). Small molecule downregulation of PmrAB reverses lipid A modification and breaks colistin resistance. ACS Chem. Biol. 9, 122–127. doi: 10.1021/cb400490k, PMID: 24131198

[ref14] Hashemi M. M. Holden B. S. Coburn J. Taylor M. F. Weber S. Hilton B.. (2019). Proteomic analysis of resistance of Gram-negative bacteria to chlorhexidine and impacts on susceptibility to colistin, antimicrobial peptides, and ceragenins. Front. Microbiol. 10:210. doi: 10.3389/fmicb.2019.00210, PMID: 30833936 PMC6388577

[ref15] Holm L. Laiho A. Törönen P. Salgado M. (2023). DALI shines a light on remote homologs: one hundred discoveries. Protein Sci. 32:e4519. doi: 10.1002/pro.4519, PMID: 36419248 PMC9793968

[ref16] Irwin J. J. Sterling T. Mysinger M. M. Bolstad E. S. Coleman R. G. (2012). ZINC: a free tool to discover chemistry for biology. J. Chem. Inf. Model. 52, 1757–1768. doi: 10.1021/ci3001277, PMID: 22587354 PMC3402020

[ref17] Kamoshida G. Akaji T. Takemoto N. Suzuki Y. Sato Y. Kai D.. (2020). Lipopolysaccharide-deficient *Acinetobacter baumannii* due to Colistin resistance is killed by neutrophil-produced lysozyme. Front. Microbiol. 11:573. doi: 10.3389/fmicb.2020.00573, PMID: 32373082 PMC7183746

[ref18] Karakonstantis S. Kritsotakis E. I. Gikas A. (2020). Pandrug-resistant gram-negative bacteria: a systematic review of current epidemiology, prognosis and treatment options. J. Antimicrob. Chemother. 75, 271–282. doi: 10.1093/jac/dkz401, PMID: 31586417

[ref19] Kramarska E. Toumi E. Squeglia F. Laverde D. Napolitano V. Frapy E.. (2024). A rationally designed antigen elicits protective antibodies against multiple nosocomial Gram-positive pathogens. NPJ Vaccines 9:151. doi: 10.1038/s41541-024-00940-x, PMID: 39155280 PMC11330964

[ref20] Lesho E. Yoon E.-J. McGann P. Snesrud E. Kwak Y. Milillo M.. (2013). Emergence of colistin-resistance in extremely drug-resistant *Acinetobacter baumannii* containing a novel pmrCAB operon during colistin therapy of wound infections. J. Infect. Dis. 208, 1142–1151. doi: 10.1093/infdis/jit293, PMID: 23812239

[ref21] Livak K. J. Schmittgen T. D. (2001). Analysis of relative gene expression data using real-time quantitative PCR and the 2(-Delta Delta C(T)) method. Methods 25, 402–408. doi: 10.1006/meth.2001.1262, PMID: 11846609

[ref22] Meng E. C. Goddard T. D. Pettersen E. F. Couch G. S. Pearson Z. J. Morris J. H.. (2023). UCSF ChimeraX: tools for structure building and analysis. Protein Sci. 32:e4792. doi: 10.1002/pro.4792, PMID: 37774136 PMC10588335

[ref23] Migliaccio A. Esposito E. P. Bagattini M. Berisio R. Triassi M. De Gregorio E.. (2021). Inhibition of AdeB, AceI, and AmvA efflux pumps restores chlorhexidine and benzalkonium susceptibility in *Acinetobacter baumannii* ATCC 19606. Front. Microbiol. 12:790263. doi: 10.3389/fmicb.2021.790263, PMID: 35197939 PMC8859242

[ref24] Mistry J. Chuguransky S. Williams L. Qureshi M. Salazar G. A. Sonnhammer E. L. L.. (2021). Pfam: the protein families database in 2021. Nucleic Acids Res. 49, D412–D419. doi: 10.1093/nar/gkaa913, PMID: 33125078 PMC7779014

[ref25] Moffatt J. H. Harper M. Harrison P. Hale J. D. F. Vinogradov E. Seemann T.. (2010). Colistin resistance in *Acinetobacter baumannii* is mediated by complete loss of lipopolysaccharide production. Antimicrob. Agents Chemother. 54, 4971–4977. doi: 10.1128/AAC.00834-10, PMID: 20855724 PMC2981238

[ref26] Novović K. Jovčić B. (2023). Colistin resistance in *Acinetobacter baumannii*: molecular mechanisms and epidemiology. Antibiotics 12:516. doi: 10.3390/antibiotics12030516, PMID: 36978383 PMC10044110

[ref27] Nurtop E. Bayındır Bilman F. Menekse S. Kurt Azap O. Gönen M. Ergonul O.. (2019). Promoters of colistin resistance in *Acinetobacter baumannii* infections. Microb. Drug Resist. 25, 997–1002. doi: 10.1089/mdr.2018.0396, PMID: 30964377

[ref28] Olaitan A. O. Morand S. Rolain J.-M. (2014). Mechanisms of polymyxin resistance: acquired and intrinsic resistance in bacteria. Front. Microbiol. 5:643. doi: 10.3389/fmicb.2014.00643, PMID: 25505462 PMC4244539

[ref29] Ouyang Z. He W. Jiao M. Yu Q. Guo Y. Refat M.. (2024). Mechanistic and biophysical characterization of polymyxin resistance response regulator PmrA in *Acinetobacter baumannii*. Front. Microbiol. 15:1293990. doi: 10.3389/fmicb.2024.1293990, PMID: 38476937 PMC10927774

[ref30] Oyejobi G. K. Olaniyan S. O. Yusuf N.-A. Ojewande D. A. Awopetu M. J. Oyeniran G. O.. (2022). *Acinetobacter baumannii*: more ways to die. Microbiol. Res. 261:127069. doi: 10.1016/j.micres.2022.127069, PMID: 35623161

[ref31] Sadones O. Kramarska E. Laverde D. Berisio R. Huebner J. Romero-Saavedra F. (2024). Investigation of cross-opsonic effect leads to the discovery of PPIase-domain containing protein vaccine candidate to prevent infections by Gram-positive ESKAPE pathogens. BMC Microbiol. 24:280. doi: 10.1186/s12866-024-03427-w, PMID: 39068414 PMC11282748

[ref32] Seleim S. M. Mostafa M. S. Ouda N. H. Shash R. Y. (2022). The role of pmrCAB genes in colistin-resistant *Acinetobacter baumannii*. Sci. Rep. 12:20951. doi: 10.1038/s41598-022-25226-x, PMID: 36470921 PMC9722906

[ref33] Stabile M. Esposito A. Iula V. D. Guaragna A. De Gregorio E. (2023). PYED-1 overcomes colistin resistance in *Acinetobacter baumannii*. Pathogens 12:1323. doi: 10.3390/pathogens12111323, PMID: 38003788 PMC10674209

[ref34] Suardíaz R. Lythell E. Hinchliffe P. van der Kamp M. Spencer J. Fey N.. (2021). Catalytic mechanism of the colistin resistance protein MCR-1. Org. Biomol. Chem. 19, 3813–3819. doi: 10.1039/D0OB02566F, PMID: 33606866 PMC8097703

[ref35] Trebosc V. Gartenmann S. Tötzl M. Lucchini V. Schellhorn B. Pieren M.. (2019). Dissecting colistin resistance mechanisms in extensively drug-resistant *Acinetobacter baumannii* clinical isolates. MBio 10, e01083–e01019. doi: 10.1128/mBio.01083-19 31311879 PMC6635527

[ref36] Vollaro A. Esposito A. Esposito E. P. Zarrilli R. Guaragna A. De Gregorio E. (2020). PYED-1 inhibits biofilm formation and disrupts the preformed biofilm of *Staphylococcus aureus*. Antibiotics (Basel) 9:240. doi: 10.3390/antibiotics9050240, PMID: 32397205 PMC7277567

[ref37] Webb B. Sali A. (2021). Protein structure modeling with MODELLER. Methods Mol. Biol. 2199, 239–255. doi: 10.1007/978-1-0716-0892-0_14 33125654

[ref38] Zafer M. M. Hussein A. F. A. Al-Agamy M. H. Radwan H. H. Hamed S. M. (2023). Retained colistin susceptibility in clinical *Acinetobacter baumannii* isolates with multiple mutations in pmrCAB and lpxACD operons. Front. Cell. Infect. Microbiol. 13:1229473. doi: 10.3389/fcimb.2023.1229473, PMID: 37600939 PMC10436201

[ref39] Zampaloni C. Mattei P. Bleicher K. Winther L. Thäte C. Bucher C.. (2024). A novel antibiotic class targeting the lipopolysaccharide transporter. Nature 625, 566–571. doi: 10.1038/s41586-023-06873-0, PMID: 38172634 PMC10794144

