# Supplementary material for: Structure-based targeting of the lipid A-modifying enzyme PmrC to contrast colistin resistance in Acinetobacter baumannii
Source: Front Microbiol. 2024 Nov 28;15:1501051. doi: 10.3389/fmicb.2024.1501051 (PMC11634806; doi:10.3389/fmicb.2024.1501051)
Supplement: Supplementary file 1 [file Data_Sheet_1.docx]

Supplementary Material

# Supplementary Figures and Tables

## Supplementary Figures


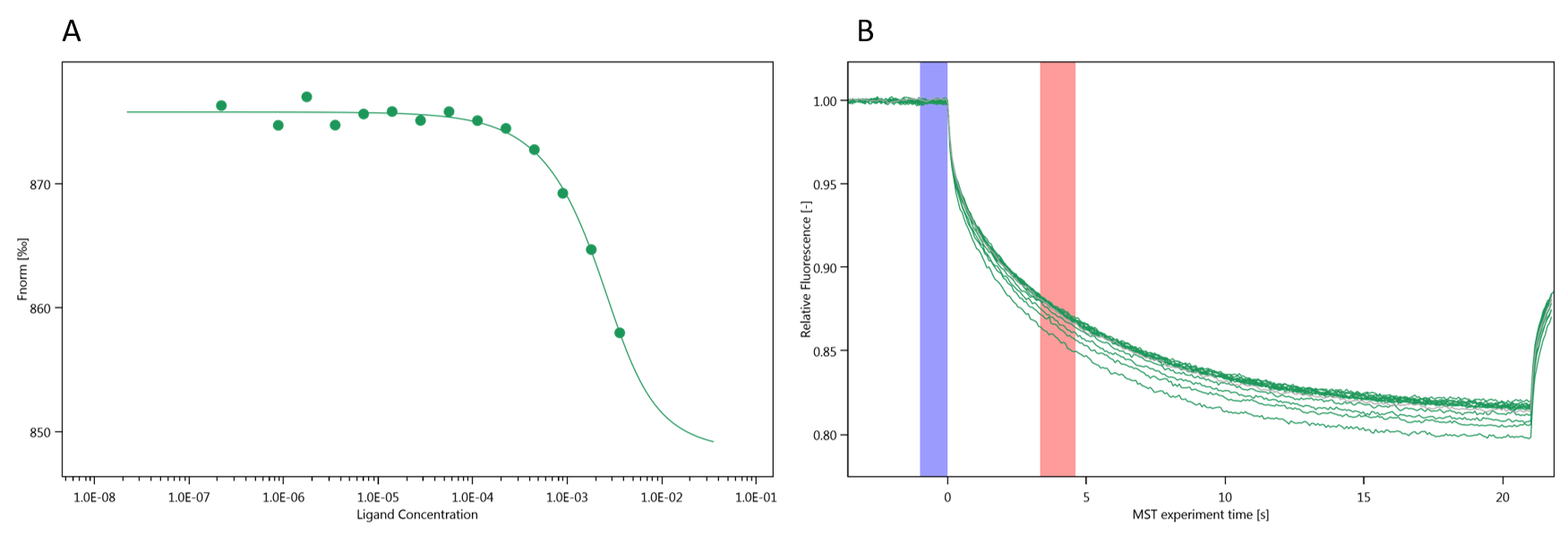


**Supplementary Figure 1.** Thermophoretic traces of MST assay for the binding to PmrC of s-Phen.

**
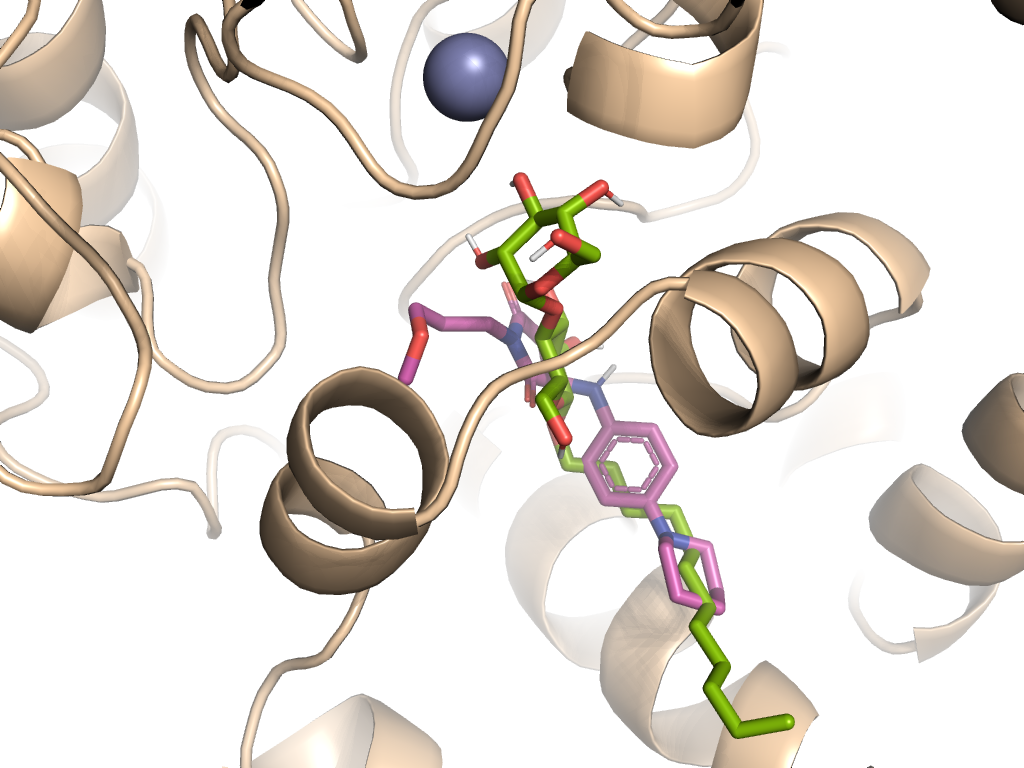
**

**Supplementary Figure 2.** Localisation of s-Phen and DDM in PmrC binding pocket after docking. S-Phen is drawn in prune stick and DDM in green stick representation and PmrC in cartoon.

**Supplementary Tables**

**Supplementary Table 1.** Structure alignment of PmrC model structure in the DALI database. Only structures in DALI 25 with Z score>10 are reported.

| **Protein name** | **PDB ID** | **Z-score** | **RMSD** | **lali** | **nres** | **%id** |
| --- | --- | --- | --- | --- | --- | --- |
| Lipooligosaccharide phosphoethanolamine transferase A (EptA) - *Neisseria meningitidis* | 5GFN | 58.0 | 0.4 | 328 | 536 | 44 |
| Endo-4-O-sulfatase - *Bacteroides thetaiotaomicron* | 6S21 | 24.7 | 3.1 | 276 | 486 | 16 |
| Sulfatase - *Akkermansia muciniphila* | 7EBP | 24.6 | 3.0 | 280 | 526 | 14 |
| Iduronate-2-sulfatase - Human | 5FQL | 24.6 | 2.8 | 276 | 507 | 18 |
| Sulfatase - *Silicibacter pomeroyi* | 4UPL | 24.6 | 2.9 | 281 | 555 | 14 |
| Putative sulfatase - *Bacteroides thetaiotaomicron* | 3B5Q | 24.3 | 3.1 | 274 | 467 | 11 |
| Sulfatase - *Bacteroides fragilis* | 6USS | 23.8 | 3.1 | 267 | 485 | 16 |
| N-sulfoglucosamine sulfohydrolase (SGSH) - Human | 4MIV | 23.2 | 3.0 | 266 | 480 | 14 |
| Steryl-sulfatase - Human | 8EG3 | 22.0 | 3.3 | 261 | 554 | 15 |
| Inner membrane protein - *Escherichia coli* | 6XLP | 21.7 | 3.0 | 256 | 586 | 13 |
| Alkaline Phosphatase (VP1736) - *Vibrio parahaemolyticus* | 3LXQ | 21.3 | 3.1 | 267 | 409 | 16 |
| Ectonucleotide pyrophosphatase/phosphodiesterase 3 - Human | 6C01 | 20.2 | 3.0 | 248 | 819 | 14 |
| Processed glycerol phosphate Lipoteichoic acid Synthase (LtaS) - *Staphylococcus aureus* | 2W5Q | 20.0 | 3.6 | 263 | 424 | 16 |
| Cellulose biosynthesis protein BcsG - *Salmonella typhimurium* | 5OLT | 20.0 | 3.2 | 260 | 383 | 11 |
| Arylsulfatase - *Pseudomonas Aeruginosa* | 4CYS | 19.9 | 3.2 | 269 | 534 | 14 |
| Alkaline phosphatase PhoK - *Sphingomonas* | 5XWK | 19.8 | 3.2 | 254 | 530 | 14 |
| Phosphoglycerol transferase GacH - *Streptococcus pyogenes* | 6DGM | 17.3 | 3.7 | 252 | 382 | 10 |
| Phosphopentomutase - *Streptococcus mutans* | 4N7T | 17.1 | 3.0 | 221 | 402 | 12 |
| Alkaline phosphatase – *Vibrio sp.* | 7QOW | 15.8 | 3.2 | 224 | 505 | 13 |
| 2,3-Bisphosphoglycerate-independent phosphoglycerate mutase - *Pyrococcus horikoshii* | 2ZKT | 15.5 | 2.9 | 210 | 381 | 10 |
| Phosphonoacetate hydrolase - *Sinorhizobium meliloti* | 3SZY | 15.4 | 3.4 | 224 | 413 | 13 |
| Cofactor-independent phosphoglycerate mutase - *Leishmania mexicana* | 3IGY | 14.1 | 3.5 | 239 | 549 | 8 |
| Acid Phosphatase A (AcpA)- *Francisella tularensis* | 2D1G | 14.4 | 3.2 | 218 | 481 | 12 |
| Non-specific phospholipase C4 - *Arabidopsis thaliana* | 8HAW | 13.7 | 3.5 | 215 | 479 | 9 |
| Intestinal-type alkaline phosphatase 1 - *Rattus norvegicus* | 4KJG | 13.7 | 3.4 | 219 | 486 | 11 |
| Alkaline phosphatase - *Thermus thermophilus* | 7KWD | 10.7 | 3.3 | 211 | 473 | 11 |

**Supplementary Table 2.** Potential PmrC inhibitors identified using virtual screening

| **ID Number** | **IUPAC name** | **Chemical Structure** | **IFDScore** | **Docking XP Gscore** |
| --- | --- | --- | --- | --- |
| F0617-0288 | 4-ethoxy-N-(2-{3-[({[4-(trifluoromethoxy)phenyl]carbamoyl}methyl)sulfanyl]-1H-indol-1-yl}ethyl)benzamide |  | -983.03 | -9.587 |
| F3305-0451 | 2-{[3-(4-chlorophenyl)-2,5-dimethylpyrazolo[1,5-a]pyrimidin-7-yl]sulfanyl}-1-(thiophen-2-yl)ethan-1-one |  | -978.77 | -9.39 |
| F2536-1922 | N-(9,10-dioxo-9,10-dihydroanthracen-2-yl)-2-(4-fluorobenzenesulfonyl)acetamide |  | -978.01 | -9.411 |
| F6473-4335 | 2-{1-[1-methyl-6-oxo-4-(thiophen-2-yl)-1,6-dihydropyridine-3-carbonyl]azetidin-3-yl}-2,3,3a,4,7,7a-hexahydro-1H-isoindole-1,3-dione |  | -977.32 | -9.382 |
| F1864-0203 | 4-[(4-benzylpiperidin-1-yl)methyl]-7-hydroxy-8-methyl-2H-chromen-2-one |  | -976.99 | -11.55 |
| F6155-0848 | N-[3-(6-oxo-1,6-dihydropyridazin-1-yl)propyl]-1-[4-(trifluoromethyl)-1,3-benzothiazol-2-yl]azetidine-3-carboxamide |  | -976.94 | -8.128 |
| F3411-0230 | 1-butyl-3-(2,5-dimethylbenzenesulfonyl)-7-(3,5-dimethylpiperidin-1-yl)-6-fluoro-1,4-dihydroquinolin-4-one |  | -976.92 | -10.242 |
| F6495-5910 | 1-[6-(3,5-dimethyl-1H-pyrazol-1-yl)pyrimidin-4-yl]-N-[4-(pyridin-2-yl)-1,3-thiazol-2-yl]azetidine-3-carboxamide |  | -976.25 | -8.609 |
| F3168-0171 | 3-{4-[4-(2,5-dimethylphenyl)piperazin-1-yl]-4-oxobutyl}-1H,2H,3H,4H-thieno[3,2-d]pyrimidine-2,4-dione |  | -975.99 | -8.035 |
| F3320-0359 | 1-(2,5-dimethylphenyl)-4-[1-(2-methoxyethyl)-1H-1,3-benzodiazol-2-yl]pyrrolidin-2-one |  | -975.92 | -8.923 |
| F1094-0179 | 3-{5-acetyl-4-methyl-3,11-dioxa-9-azatetracyclo[11.4.0.0^{2,6}.0^{7,12}]heptadeca-1(13),2(6),4,7(12),14,16-hexaen-9-yl}-1lambda6-thiolane-1,1-dione |  | -975.84 | -9.456 |
| F1681-0035 | 1-(2-{2-hydroxy-3-[4-(4-methoxyphenyl)piperazin-1-yl]propoxy}phenyl)ethan-1-one dihydrochloride |  | -974.79 | -10.511 |
| F5485-0149 | 2-[2-(benzylsulfanyl)-6-oxo-1,6-dihydropyrimidin-4-yl]-N-(3-acetamidophenyl)acetamide |  | -994.71 | -11.979 |
| F0394-0044 | 4-[5-(3-bromophenyl)-3-(6-chloro-2-oxo-4-phenyl-1,2-dihydroquinolin-3-yl)-4,5-dihydro-1H-pyrazol-1-yl]-4-oxobutanoic acid |  | -980.57 | -9.187 |
| F0770-0058 | 5-[3-(6-bromo-2-oxo-4-phenyl-1,2-dihydroquinolin-3-yl)-5-(2-chlorophenyl)-4,5-dihydro-1H-pyrazol-1-yl]-5-oxopentanoic acid |  | -980.31 | -10.895 |
| F6472-6100 | 2-oxo-6-{2-[4-(propan-2-ylsulfanyl)phenyl]acetyl}-1,2,5,6,7,8-hexahydro-1,6-naphthyridine-3-carbonitrile |  | -979.33 | -10.072 |
| F3115-0081 | N-{5-[(benzylcarbamoyl)methyl]-3-(4-fluorophenyl)-2,4-dioxoimidazolidin-1-yl}benzamide |  | -978.95 | -9.563 |
| F3205-0035 | 1-(3-methoxypropyl)-3-{[4-(piperidin-1-yl)phenyl]amino}pyrrolidine-2,5-dione |  | -976.65 | -9.605 |
| F2334-0542 | 2-[(2,5-dimethylphenyl)methyl]-8-(4-methylphenyl)-2H,3H,4H,6H,7H,8H-imidazo[2,1-c][1,2,4]triazine-3,4-dione |  | -973.72 | -9.408 |
| F0158-0023 | 2-(3-oxo-1,2,3,4-tetrahydroquinoxalin-2-yl)-N-[2-(trifluoromethyl)phenyl]acetamide |  | -973.58 | -11.081 |
| F6761-7637 | 6-{5-[4-(trifluoromethyl)pyridin-2-yl]-octahydropyrrolo[3,4-b]pyrrole-1-carbonyl}-2,3-dihydropyridazin-3-one |  | -970.0 | -8.193 |
| F3225-6988 | 2-(2-fluorophenyl)-3-[(furan-2-yl)methyl]-10-methyl-3H,4H,5H,10H-pyrimido[4,5-b]quinoline-4,5-dione |  | -990.68 | -8.229 |
| F6523-3945 | 13-fluoro-5-(2-hydroxyquinoline-4-carbonyl)-1,5,9-triazatricyclo[8.4.0.0^{3,8}]tetradeca-3(8),9,11,13-tetraen-2-one |  | -987.61 | -9.081 |
| F6548-3997 | N-(4-ethylphenyl)-2-{[2-({[(4-fluorophenyl)methyl]carbamoyl}methyl)-3-oxo-2H,3H-imidazo[1,2-c]quinazolin-5-yl]sulfanyl}butanamide |  | -986.29 | -7.962 |
| F6231-0947 | N-{2-[3-(2-methyl-1H-imidazol-1-yl)-6-oxo-1,6-dihydropyridazin-1-yl]ethyl}-9H-xanthene-9-carboxamide |  | -974.35 | -9.381 |
| F6424-0643 | N-[2-(1-methyl-6-oxo-1,6-dihydropyridazin-3-yl)phenyl]-3-(trifluoromethyl)benzamide |  | -972.22 | -11.582 |

**Supplementary Table 3.** Sequence identities (%) of PetN transferases with PmrC of *A. baumannii*

| **Protein** | **PmrC** | **PmrC1** | **EptA** | **EptA-1** | **EptA-2** |
| --- | --- | --- | --- | --- | --- |
| **PmrC** | 100 | 84.9 | 97.1 | 89.1 | 89.8 |
| **PmrC1** |  | 100 | 87.8 | 81.0 | 81.3 |
| **EptA** |  |  | 100 | 90.0 | 90.7 |
| **EptA-1** |  |  |  | 100 | 97.1 |
| **EptA-2** |  |  |  |  | 100 |

Alignments were performed using EMBOSS Needle using sequences: PmrC, A. baumannii AB0057 PmrC; PmrC1, A. baumannii MRSN3405 PmrC1; EptA, A. baumannii MDR-TJ EptA; EptA-1, A. baumannii MRSN3405 EptA homolog; EptA-2, A. baumannii MRSN3405 EptA homolog;

**Supplementary Table 4**. Forward and reverse primers for *pmrA*, *pmrB*, *pmrC*, and *16S rRNA* used for qRT-PCR

| Gene | Forward Primer (5’-3’) | Reverse Primer (5’-3’) | Reference |
| --- | --- | --- | --- |
| *pmrA* | ATGACAAAAATCTTGATGATTGAAGAT | CCATCATAGGCAATCCTAAATCCA | (1) |
| *pmrB* | GAACAGCTGAGCACCCTTTAA | ACAGGTGGAACCAGCAAATG | (1) |
| *pmrC* | CTCTTTACGCTTTGTTTTATGGAC | GTAAAAAGTAAAACACCGACCA | (1) |
| *16S rRNA* | TCAGCTCGTGTCGTGAGATG | CGTAAGGGCCATGATG | (1) |

(1). Pournaras S, Poulou A, Dafopoulou K, Chabane YN, Kristo I, Makris D, Hardouin J, Cosette P, Tsakris A, Dé E. Growth retardation, reduced invasiveness, and impaired colistin-mediated cell death associated with colistin resistance development in Acinetobacter baumannii. Antimicrob Agents Chemother. 2014;58(2):828-32.
